# Further refinement of high standards of care– focus on polytrauma

**DOI:** 10.1007/s00068-024-02543-6

**Published:** 2024-05-21

**Authors:** Philipp Störmann

**Affiliations:** Department of Trauma Surgery and Orthopedics, University Hospital Goethe University Frankfurt, Theodor-Stern-Kai 7, D-60590 Frankfurt / Main, Germany

Enormous progress has been made in polytrauma care over the last two decades. Nevertheless, the optimal care of this highly critical patient group remains challenging and continues to be one of the central components of current trauma research. There are unanswered questions in all phases of treatment, and the seven articles in this focus on polytrauma issue in the European Journal of Trauma and Emergency Surgery shed light on several aspects.

Polytrauma is no longer perceived as a purely anatomical injury, but as an overall picture of the individual injuries, acute pathophysiology, and the induced inflammatory reaction with the interference between many organ systems. The differentiation between severe isolated trauma (Injury Severity Score > 15) and polytrauma is therefore of special importance, as multiple injuries usually result in a much more distinct remote organ injury. In their prospective Australian study, Hardy et al. show that the overall incidence of severe trauma is continuously increasing. Although the number of polytrauma patients remained roughly the same, mortality in this group fell significantly between 2009 and 2021, confirming the progress made in the care of this highly complex group of patients [1].In order to establish and further refine the already high standard of trauma care described above throughout Europe, Hietbrink et al. [2] carried out a comprehensive analysis of the necessary requirements for a surgeon-based and patient-centered care of polytrauma patients in their manuscript. They prompt, that severe trauma should be regarded as a surgical disease and the trauma surgeon should be (recognized as) a specialist who has knowledge of both physiology, injuries and life-saving surgical procedures.

Intensive discussions are currently underway to achieve the most precise activation of the trauma team (trauma team activation - TTA). It is apparent that the existing triage protocols often do not identify the polytraumatized patient with sufficient accuracy. In their retrospective analysis, Hagebusch et al. [3] determined serum lactate levels in patients in whom TTA was performed solely due to the mechanism of trauma. They were able to show that lactate levels, especially in older patients, are very suitable for identifying patients with an ISS > 15 and suggest that lactate should be included in triage algorithms to reduce undertriage. In the early clinical care phase, the comprehensive and complete diagnosis of all existing injuries with the initial stabilization of the patient is the main priority in polytrauma care. The performance of a trauma scan has clearly emerged as the gold standard. There is no doubt that a proximity of the trauma bay to the CT scanner is advantageous for the patient, if only because of the time advantage. In their study, Lucas et al. examined the next generation of CT, the sliding gantry which is located directly in the trauma room. They were able to demonstrate a further time advantage for this constellation compared to the conventional arrangement, regardless of the severity of the injury. However, they recommend regular training of the trauma teams in order to avoid practical problems [4].

Scoring systems have always played a decisive role in polytrauma to classify the quality of care, to describe the severity of injury as accurately as possible and also for research purposes. As new scores are regularly added to the numerous existing ones, Girshausen et al. [5] compared well-established scores with newer ones in this study. They were able to show that the RISC II score has the greatest predictive capability for mortality, even in comparison with the frequently used but simpler scores such as the ISS. One major focus of research in recent years has been the strategic treatment of major fractures in polytrauma. The main focus here has been on planning the optimum time for the final treatment of e.g. femoral shaft fractures, in order to minimize the overall inflammatory burden. Despite all the research in this field, the term *major fracture* is not clearly defined, so the Polytrauma Section of the ESTES is attempting to clarify this term as part of a literature search in their recent manuscript. Kalbas et al. [6] show that major fractures have had a noticeable influence on treatment strategies in recent times. In the last decade, the research focus has increasingly shifted from the treatment of femoral fractures to pelvic and spinal fractures. As previously mentioned, the timing of optimal treatment of major fractures in polytraumatized patients plays a pivotal role. In the field between damage control surgery and safe definitive surgery, not only the physiology of the patient but also special features of the regional trauma systems appear to play a role. In order to shed light on these differences, Scherer et al. conducted a Europe-wide survey on the care of polytrauma patients. This showed that the participants use different definitions of polytrauma (e.g. ISS < 15; “Berlin definition”). Normal coagulation, the absence of vasopressors as well as the absence of clinical signs of SIRS are most frequently described as criteria for performance of safe definitive surgery [7]. The working group thus continues to describe the shift from a defined window of opportunity to a more patient-centered approach in fracture treatment based on physiological values.

This Focus on Polytrauma issue of the EJTES offers exciting new insights into diagnostic and treatment of polytraumatized patients and also deals with transnational discussion points in Europe. I hope you enjoy reading the exciting articles.

## References

[1] Hardy BM, King KL, Enninghorst N, Balogh ZJ. Trends in polytrauma incidence among major trauma admissions. Eur J Trauma Emerg Surg. 2022. 10.1007/s00068-022-02200-w PMC11249606 36536173

[2] Hietbrink F, Mohseni S, Mariani D, Naess PA, Rey-Valcárcel C, Biloslavo A et al. What trauma patients need: the European dilemma. Eur J Trauma Emerg Surg. 2022. 10.1007/s00068-022-02014-w PMC11249462 35798972

[3] Hagebusch P, Faul P, Ruckes C, Störmann P, Marzi I, Hoffmann R et al. The predictive value of serum lactate to forecast injury severity in trauma-patients increases taking age into account. Eur J Trauma Emerg Surg. 2022. 10.1007/s00068-022-02046-2 35852548

[4] Lucas B, Meng M, Schirrmeister W, Pliske G, Walcher F, Schüttrumpf JP. Lessons learned during the sliding gantry CT implementation in a trauma suite. Eur J Trauma Emerg Surg. 2022. 10.1007/s00068-022-02080-0 PMC11249429 35988107

[5] Girshausen R, Horst K, Herren C, Bläsius F, Hildebrand F, Andruszkow H. Polytrauma scoring revisited: prognostic validity and usability in daily clinical practice. Eur J Trauma Emerg Surg. 2022. 10.1007/s00068-022-02035-5 PMC11249471 35819474

[6] Kalbas Y, Klingebiel FKL, Halvachizadeh S, Kumabe Y, Scherer J, Teuben M et al. Developments in the understanding of staging a major fracture in polytrauma: results from an initiative by the polytrauma section of ESTES. Eur J Trauma Emerg Surg. 2023. 10.1007/s00068-023-02245-5 PMC11249440 36820896

[7] Scherer J, Coimbra R, Mariani D, Leenen L, Komadina R, Peralta R et al. Standards of fracture care in polytrauma: results of a Europe-wide survey by the ESTES polytrauma section. Eur J Trauma Emerg Surg. 2022. 10.1007/s00068-022-02126-3 PMC11249422 36227354

